# Identification and Prognostic Analysis of Immune-Related Genes Co-Regulated by Key Histone Modifications in Breast Cancer

**DOI:** 10.3390/cimb48060582

**Published:** 2026-06-01

**Authors:** Yanni Cao, Xiaohui Li, Jiangshan Liu, Junyuan Zhang, Kangcheng Xu, Hao Lin, Yuxian Liu

**Affiliations:** 1 School of Artificial Intelligence, Anhui University of Science & Technology, Huainan 232001, China; cyn@aust.edu.cn (Y.C.);; 2 State Key Laboratory of Digital Intelligent Technology for Unmanned Coal Mining, Anhui University of Science & Technology, Huainan 232001, China; 3 Center for Informational Biology, School of Life Sciences and Technology, University of Electronic Science and Technology of China, Chengdu 611731, China

**Keywords:** histone modification, immune-related genes, risk scoring model, immune infiltration, drug sensitivity, biomarkers

## Abstract

Background: Breast cancer (BRCA) is a common malignant tumor that seriously threatens women’s health. Studies have shown that histone modifications (HMs) play a vital role in the occurrence and development of BRCA. This study aims to explore the distribution patterns of HMs in the mammary epithelial cell line (HMEC) and breast cancer cell line (MCF-7), and their potential associations with gene expression, patient prognosis, and drug efficacy. Methods: First, the distribution of histone modification (HM) signals in HMEC and MCF-7 cell lines was analyzed. Multiple algorithms were then used to predict the effects of different HMs and their modified regions on gene expression in the two cell lines. Based on four key regions identified from this analysis, 268 HM-related immune-related genes (H_IRGs) were screened, followed by functional enrichment and pathway analysis. Subsequently, Cox and LASSO regression analyses were performed on the H_IRGs to construct a risk scoring model. Results: The random forest algorithm showed the best predictive performance (AUC = 0.92) and identified three key HMs (H3K4me2, H3K27me3, and H3K36me3) and four key regions that strongly influenced gene expression. A risk scoring model was constructed from 11 key IRGs (*BCL2A1*, *PSME2*, *STC2*, *ESRRG*, *CRISP3*, *IL13RA1*, *LCN1*, *EED*, *CLEC10A*, *SLURP1*, and *FGF12*). This model effectively predicted patients’ survival in both the training and validation cohorts. Conclusions: In summary, our research results provide a theoretical basis for the occurrence and development of BRCA, and the 11 key IRGs discovered are expected to become potential biomarkers for BRCA prognostic assessment and treatment response prediction.

## 1. Introduction

Breast cancer (BRCA) is the most common malignant tumor among women worldwide, and its incidence has been rising globally in recent years. According to statistics from the International Agency for Research on Cancer (IARC), BRCA has become a major factor contributing to the global cancer burden [1]. Taking the United States as an example, it was expected that there would be 319,750 new cases and 42,680 related deaths in 2025, highlighting the severity of the challenge [2]. With the advancement of medicine, the application of comprehensive treatment methods such as neoadjuvant chemotherapy, targeted therapy and immunotherapy have significantly improved the prognosis of patients with early-stage BRCA [3], and new diagnostic and treatment technologies have further promoted the improvement of patients’ survival outcomes [4,5]. However, the mortality rate of BRCA patients is still very high today. In-depth analysis of the molecular mechanism of BRCA occurrence and development [6], and exploration of biomarkers and therapeutic targets with clinical application value, are still the important directions of current research.

The occurrence, development, and metastasis of tumors involve complex networks of molecular regulatory systems, and epigenetic mechanisms play a key regulatory role in this process [7,8,9,10,11]. Compared with the irreversible changes of traditional gene sequences, epigenetic regulation, such as DNA methylation and histone modifications (HMs), has dynamic and reversible characteristics, which provides unique advantages for the development of targeted therapeutic drugs [12,13,14,15,16]. HM is an important epigenetic modification that is closely related to gene expression [17,18,19]. It mainly regulates gene expression by changing chromatin structure and recruiting specific proteins [20,21]. More and more studies have shown that HMs are deeply involved in the occurrence and development of BRCA and play an important role in the regulation of gene expression. For example, increased H3K27ac levels in BRCA can promote the expression of *SOX4*, thereby promoting cancer cell proliferation and invasion, and the formation of related lymphatic vessels [22]. Increased H3K4me1 levels can activate the expression of genes related to the c-Myc/*SENP1* pathway, thereby exacerbating the metastasis of triple-negative breast cancer (TNBC) and leading to poor prognosis for patients [23]. Downregulation of *KDM5A* expression leads to a decrease in H3K4me3 levels, thereby promoting the expression of the tumor suppressor gene *p16*, effectively inhibiting the occurrence and metastasis of TNBC [24]. It is worth noting that HMs exhibit a bidirectional regulatory effect on gene expression. H3K9me3 loss promotes the transcription of oncogenes [25], while reducing H3K36me3 levels inhibits the expression of *PHGDH* [26]. In addition, studies have found that increased levels of H3K4me3 and H3K9ac are associated with shortened survival in BRCA patients [27], while increased levels of H4K20me3, H3K4me2, and H3K9me2 are associated with a good prognosis [28]. Although existing studies have confirmed that HMs play a key role in the regulation of BRCA gene expression, their specific regulatory mechanism remains unclear, which provides a key breakthrough for the development of epigenetic targeting therapeutic strategies.

With the rise of cancer immunotherapy, the application of immune-related genes (IRGs) in the treatment of BRCA has received widespread attention [29,30,31]. Studies have found that some IRGs are closely related to the prognosis of BRCA patients. For example, the complex signaling mechanism of *IL6ST* plays a key role in immunology and various cancers. In TNBC, higher *IL6ST* levels are significantly associated with better overall survival of patients [32]. Upregulation of *CTLA-4* and *TIGIT* expression indicates poor prognosis in BRCA patients [33]. *ATP2C2* is an emerging immune-related biomarker, and low expression of it indicates a good prognosis for TNBC patients [34]. In addition, more and more studies have shown that the prognostic model based on IRGs can better support the prognostic assessment of BRCA patients. The risk model established by five prognosis-related IRGs has AUC values of 0.791 and 0.859 for predicting TNBC patients’ 3 years and 5 years outcomes, respectively. The expression of these genes in TNBC tissues is consistent with the immunohistochemistry results, showing good predictive performance [35]. The study of luminal B breast cancer has demonstrated that a 12-gene prognostic IRG model can effectively predict the clinical outcomes of BRCA patients, and that these genes are closely related to tumor progression and immunotherapy [36]. Therefore, this study selected IRGs as the main target for subsequent research to provide new insights into BRCA treatment.

In this work, using machine learning and bioinformatics methods, we first analyzed the distribution of HM signals in HMEC and MCF-7 cells. We then used multiple algorithms, including random forests [37], to predict upregulated and downregulated genes and identified four key modified regions of three key HMs that co-regulate gene expression. Additionally, enrichment analysis of 268 H_IRGs within these key regions confirmed their involvement in cancer development and progression. Furthermore, we constructed a risk scoring model to explore the specific impact of 11 key IRGs on patient prognosis. Finally, immune infiltration and drug sensitivity analyses demonstrated that these 11 key IRGs could serve as potential biomarkers for BRCA, providing a theoretical basis for BRCA therapy.

## 2. Materials and Methods

### 2.1. Data Collection and Preprocessing

First, we obtained whole-genome polyA+ RNA-seq data of the mammary epithelial cell line (HMEC) and breast cancer cell line (MCF-7) from the ENCODE database (https://www.encodeproject.org/, accessed on 22 December 2021), as well as data on 11 histone modifications (HMs) [38,39]. Subsequently, we used BEDTools (version 2.29.2) to convert the original BAM file into a BED file to facilitate subsequent visual analysis [40]. Next, we downloaded the human reference genome annotation file (RefSeq genes (hg38)) from the UCSC database (http://genome.ucsc.edu/, accessed on 22 December 2021). We excluded non-coding genes, removed duplicates based on identical transcription start sites (TSSs), and deduplicated gene names, ultimately retaining 19,293 genes for subsequent analyses. We also downloaded immune-related genes (IRGs) from the ImmPort database (https://www.immport.org/home, accessed on 22 December 2021).

Next, we downloaded transcriptome and clinical data for 1231 breast samples from the TCGA database (https://portal.gdc.cancer.gov/, accessed on 3 July 2024). We retained only frozen tissue samples. Among them, samples with the suffix “01A” are BRCA samples, and those with the suffix “11A” are normal breast samples. We finally obtained FPKM data for 1081 cancer samples and 99 normal samples, and then normalized them using the “scale” function. For the clinical data of BRCA samples, we excluded samples with overall survival greater than 15 years or less than 0 year, as well as samples with stages of unknown and X, ultimately selecting 1045 BRCA samples with complete clinical information for subsequent analysis.

Finally, we downloaded transcriptome and clinical data from the GEO database (https://www.ncbi.nlm.nih.gov/geo/, accessed on 4 March 2025) for the GSE20711 (88 BRCA samples), GSE48390 (81 BRCA samples), and GSE25055 (310 BRCA samples). When processing gene expression data from these three datasets, we used the average of replicate gene expression values as the gene expression data and performed “scale” normalization.

### 2.2. Calculation of Differentially Expressed Genes

First, the DEGseq package (version 3.58.1) was used to match the reads in the bed file to the genes, and the expression level (RPKM) value of each gene was calculated according to Formula (1) [41].

(1) $${\mathrm{RPKM}}_{i}=\frac{C_{i}\times 10^{9}}{L_{i}\times N}$$

where i represents the i-th gene, $C_{i}$ represents the total number of reads matching the exon of the i-th gene, $L_{i}$ represents the length of the exon of the i-th gene, N is the sequencing depth, and ${\mathrm{RPKM}}_{i}$ represents the expression level of the i-th gene.

Second, the DEGseq2 package (version 1.28.1) was used to identify differentially expressed genes between HMEC and MCF-7 cell lines, with a threshold of $|log_{2}FC|>1$ and adjusted *p* value (padj)<0.001.

### 2.3. Calculation of Histone Modification Levels

To calculate the signal distribution of HMs in HMEC and MCF-7 cell lines, we divided the region 10,000 bp upstream and 2000 bp downstream of TSS into 60 bins of 200 bp each (Table S1). Then, the reads in the bed file were matched to these 60 bins, and the HMs signal value of each bin was calculated according to Formula (2) [42].

(2) $$H_{i,m}^{j}=\frac{n_{i,m}^{j}\times 10^{9}}{n^{j}\times l_{m}}(1\leq i\leq 19,293,1\leq m\leq 60,1\leq j\leq 11)$$

where j represents the j-th HM, i represents the i-th gene, m represents the m-th bin, $n_{i,m}^{j}$ represents the total number of reads of the j-th HM matching to the m-th bin of the i-th gene, $n^{j}$ is the sequencing depth of the j-th HM, and $l_{m}$ represents the length of the m-th bin, and $10^{9}$ is used to maintain the same amplitude as RPKM.

### 2.4. Prediction of Upregulation and Downregulation of Gene Expression

To explore the effects of HMs on gene expression regulation, we first calculated SDHMs (HMs signal differences, $log_{2}FC$) between HMEC and MCF-7 cell lines according to Formula (3).

(3) $$log_{2}FC=log_{2}[H_{T}/(H_{N}+10^{-9})+1]$$

where $H_{T}$ is the HM signals of the MCF-7 cell line, and $H_{N}$ is the HM signals of the HMEC cell line.

Then, using SDHMs as features, 12 algorithms (Table S2) were used to predict upregulation and downregulation of BRCA gene expression. To ensure the reliability of the results, a ten-fold crossover method was used to reduce the prediction error. Finally, the area under the receiver operating characteristic curve (AUC) was obtained by calculating sensitivity ($S_{n}$) and specificity ($S_{p}$) to assess the effect of HMs on gene expression regulation [43].

(4) $$S_{n}=N^{D}/(N^{D}+N_{-U}^{D}),S_{p}=N^{U}/(N^{U}+N_{-D}^{U})$$

where $N^{D}$ represents the total number of genes correctly predicted to be downregulated (D), $N_{-U}^{D}$ represents the total number of genes incorrectly predicted to be upregulated (U) but are actually downregulated, $N^{U}$ represents the total number of genes correctly predicted to be upregulated, and $N_{-D}^{U}$ represents the total number of genes incorrectly predicted to be downregulated but are actually upregulated.

### 2.5. Enrichment Analysis of Immune-Related Genes

To explore the biological functions of BRCA-related IRGs, we used the clusterProfiler package (version 4.10.0) to perform Gene Ontology (GO) functional enrichment analysis and Kyoto Encyclopedia of Genes and Genomes (KEGG) pathway enrichment analysis, and used the Coexpedia database (http://www.coexpedia.org, accessed on 10 October 2025) [44] to perform Disease Ontology (DO) enrichment analysis, so as to gain a deeper understanding of the specific functions of these IRGs.

### 2.6. Construction and Prognostic Validation of Risk Scoring Models

To construct the IRG-based risk scoring model, we used samples from the TCGA database as a training cohort and samples from the GEO database as an external validation cohort. Then, we sequentially applied univariate Cox regression analysis, LASSO regression, and multivariate Cox regression analysis (survival package (version 3.5.1), glmnet package (version 4.1.8)), based on the multivariate Cox regression coefficients; we constructed a risk scoring model consisting of 11 key IRGs, as shown in Formula (5):

(5) $$RS=\sum_{i=1}^{11}\beta_{i}\times Expr_{i}$$

where i represents the i-th gene, $\beta_{i}$ represents the multivariate Cox regression coefficient of the i-th gene, $Expr_{i}$ represents the expression value of the i-th gene, and RS represents the patient’s risk score.

Based on the above model, we calculated the median risk score and divided the patients into high- and low-risk groups. Then we compared the survival status between the two risk groups using Kaplan–Meier survival analysis. Meanwhile, the prognostic accuracy of the risk scoring model was assessed using the receiver operating characteristic (ROC) curve over time.

### 2.7. Comparison of Immune Status

To explore the differences in immune status between high- and low-risk BRCA patients, we obtained 28 immune cell types from the TISIDB database (http://cis.hku.hk/TISIDB/, accessed on 27 October 2024) and 13 immune-related signaling pathways from related studies [45]. Subsequently, the single-sample gene set enrichment analysis method (ssGSEA, GSVA package (version 1.50.1)) was used to calculate the enrichment scores of immune cells and immune-related pathways to evaluate the differences in immune cell infiltration levels and the activities of 13 immune-related signaling pathways between high- and low-risk groups.

### 2.8. Drug Sensitivity Analysis

When using the pRRophetic package (version 0.5) for drug sensitivity analysis, we first identified commonly used chemotherapy drugs for the treatment of BRCA. Then, we combined the genomics of drug sensitivity in cancer (GDSC) data and known gene expression profiles to construct a linear regression model to predict drug sensitivity results (half-inhibitory concentration (IC50)), and then evaluated the sensitivity of different chemotherapy drugs to BRCA patients.

## 3. Results

### 3.1. Analysis of Histone Modification Distribution Patterns of Co-Differentially Expressed Immune-Related Genes

**Figure 1:**
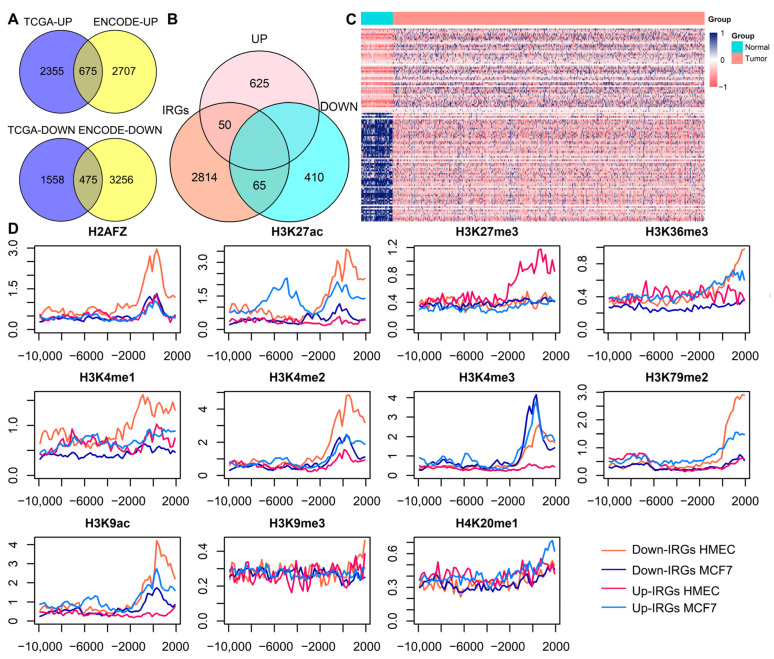
Distribution patterns of co-differentially expressed IRGs and their HM signals in HMEC and MCF-7. (**A**) Intersection of upregulated or downregulated genes in the TCGA and ENCODE databases. (**B**) Intersection of co-differentially expressed upregulated or downregulated genes and IRGs. (**C**) Heatmap of 115 co-differentially expressed IRGs between normal and cancer samples. (**D**) Distribution of HM signals of co-differentially expressed IRGs in HMEC and MCF-7. (The horizontal axis is 10,000 bp upstream and 2000 bp downstream of the TSS, and the vertical axis is the HM signal value $H_{i,m}^{j}$). Abbreviations: UP: upregulated genes, DOWN: downregulated genes, IRGs: immune-related genes.

To explore the differences in histone modification (HM) signal distribution patterns and immune-related gene (IRG) expression status between the mammary epithelial cell line (HMEC) and breast cancer cell line (MCF-7), We first analyzed gene expression data for the two cell lines downloaded from the ENCODE database. By calculating differentially expressed genes, we identified a total of 3382 upregulated genes and 3731 downregulated genes. Concurrently, we identified differentially expressed genes between normal breast tissue samples and breast cancer (BRCA) samples downloaded from the TCGA database, identifying 3030 upregulated genes and 2033 downregulated genes. Subsequently, we intersected the upregulated and downregulated genes from the two datasets (Figure 1A), and identified 675 commonly upregulated genes and 475 commonly downregulated genes. Given the important role of IRGs in tumor immunotherapy, and the ability of their modified cells to accurately identify cancer cells without damaging normal tissues, we further performed an intersection analysis of these co-expressed genes and IRGs (Figure 1B). Ultimately, we obtained 50 upregulated IRGs and 65 downregulated IRGs, and the expression values of these 115 co-differentially expressed IRGs showed significant differences between normal and cancer samples (Figure 1C).

Next, we analyzed the HM signal distribution of differentially expressed IRGs in the two cell lines (Figure 1D). The results showed that, for the downregulated IRGs, the modification levels of H2AFZ, H3K27ac, H3K36me3, H3K4me1, H3K4me2, H3K79me2, and H3K9ac were significantly higher in HMEC than in MCF-7. Conversely, for the upregulated IRGs, the modification levels of H3K27ac, H3K4me2, H3K4me3, H3K79me2 and H3K9ac were significantly higher in MCF-7 than in HMEC. Furthermore, regardless of whether the IRGs were upregulated or downregulated, the modification level of H3K4me3 in MCF-7 was significantly higher than in HMEC. In summary, the HM levels of IRGs with different expression states were significantly different in the two cell lines, indicating that the signals distribution pattern of HMs in the two cell lines are closely related to the expression state of IRGs and may affect the occurrence and development of BRCA.

### 3.2. Prediction of Upregulation and Downregulation of Gene Expression by Different HMs

**Figure 2:**
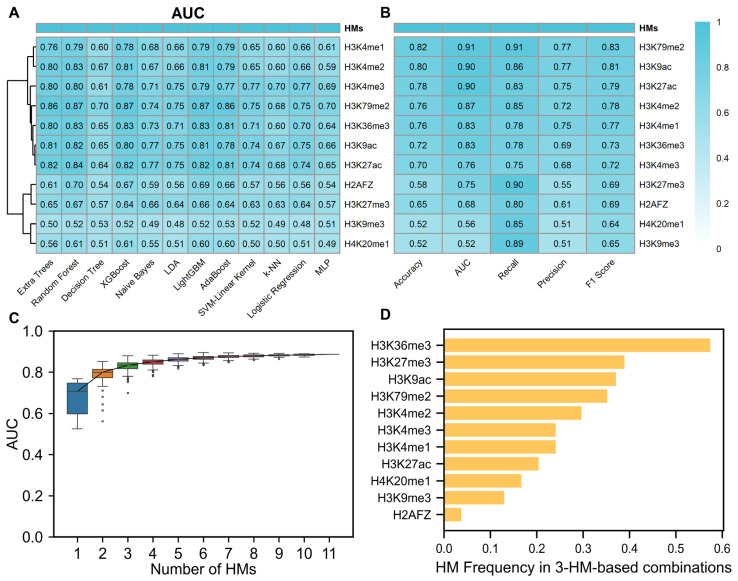
Effects of different histone modifications on gene expression. (**A**) AUC values of 11 HMs predicted by 12 algorithms in the test set. (**B**) Performance of 11 HMs predicted by the random forest algorithm for various gene expression metrics in the validation set. (**C**) AUC values of different HM combinations for predicting gene expression. (**D**) Frequency of 11 HMs with a 3-HM combination greater than the 95% AUC of the 11-HM combination. Abbreviations: HMs: histone modifications, AUC: area under the receiver operating characteristic curve.

To investigate the regulatory effects of different HMs on gene expression, we used the SDHMs of 60 bins in each HM as features, and used the co-differentially expressed genes without IRGs as the training set and the co-differentially expressed IRGs as the test set. First, we used 12 algorithms to predict the upregulation and downregulation of gene expression, using the SDHMs of 60 bins in each HM as independent features. The analysis found that the random forest algorithm had the best prediction performance (Figure 2A), and the AUC values of each HM obtained by this algorithm were generally higher than those of other algorithms. Among them, H3K4me2, H3K4me1, H3K79me2, H3K36me3, H3K27ac, and H3K9ac had relatively better predictive performance, showing their independent regulatory ability on gene expression. Subsequently, the trained random forest model was tested using a test set (Figure 2B). Among the 11 HMs, the AUC values of H3K79me2, H3K9ac, H3K27ac, H3K4me2, H3K4me1, and H3K36me3 were all greater than 0.8. This further indicates that these six HMs have a strong independent regulatory effect on gene expression.

Studies have shown that combinations of multiple HMs have a stronger regulatory effect on gene expression [46]. To identify combinations of HMs that can co-regulate gene expression, we randomly combined 11 HMs and constructed 2047 random forest models to predict upregulation and downregulation of gene expression (Figure 2C). The results showed that as the number of HMs increased, the overall prediction performance initially increased and then slowly decreased. Subsequently, we analyzed the optimal prediction results for each combination number (Table 1) and found that when the combination number was three, the AUC value of the prediction result did not improve significantly even if the number of HMs increased. Based on the consideration of model overfitting and the Occam’s razor principle, we selected the best 3-HM combination (H3K36me3, H3K27me3, H3K4me2) for subsequent analysis, which was consistent with the conclusion that H3K36me3 and H3K4me2 had better prediction performance in single modification prediction. To further clarify the importance of H3K36me3, H3K27me3, and H3K4me2 in combination, we used 95% of the AUC value of the 11-HM combination as the screening threshold and selected 54 combinations from 165 HM combinations. Among these combinations, H3K36me3 and H3K27me3 appear most frequently, and H3K4me2 is also among the top five (Figure 2D). The above results indicate that H3K36me3, H3K27me3, and H3K4me2 may be the key HM combination that co-regulates gene expression during the occurrence and development of BRCA.

### 3.3. Screening of Key Regions of Critical HMs

**Figure 3:**
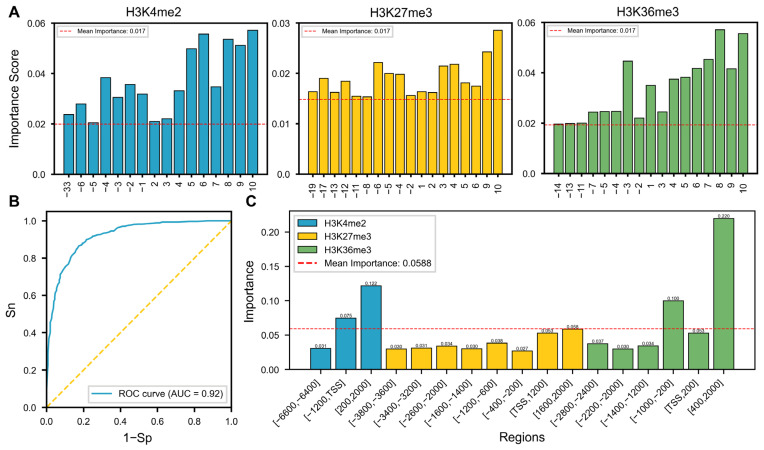
Effects of HMs in different regions on gene expression. (**A**) Screening of important bins in three HMs. (**B**) ROC curves of SDHMs for predicting gene expression in 17 important regions. (**C**) Screening of four key regions in three HMs. Abbreviations: SDHMs: HM signal differences, Sn: sensitivity, Sp: specificity.

To further explore the regulatory mechanisms of key HMs on gene expression during the development of BRCA, we studied the impact of different regions of key HMs on gene expression. First, we used the SDHMs of 60 bins from three key HMs as input features for the random forest model to predict upregulation and downregulation of gene expression. Then, the average feature importance score of 60 bins in each HMs was used as the threshold for selecting significant bins (Figure 3A), and consecutive bins were merged to obtain 17 important regions (Table 2). Next, we used the SDHMs of these regions as features to predict upregulation and downregulation of gene expression using a random forest model. The results showed that, compared with the optimal AUC in the 3-HM combination, using SDHMs in 17 important regions of the three key HMs for prediction significantly improved performance, with an AUC value of 0.92 (Figure 3B). Finally, using the average importance score of the 17 regions as a threshold, four key regions were further screened (Figure 3C), which are [−1200, TSS] bp and [200, 2000] bp for H3K4me2, and [−1000, −200] bp and [400, 2000] bp for H3K36me3. Given that H3K4me2 and H3K36me3 perform well in both single modification and combined modification prediction, this further indicates that the SDHMs in these four key regions are jointly involved in regulating gene expression.

### 3.4. Identification and Functional Analysis of HM-Related Immune Genes (H_IRGs)

**Figure 4:**
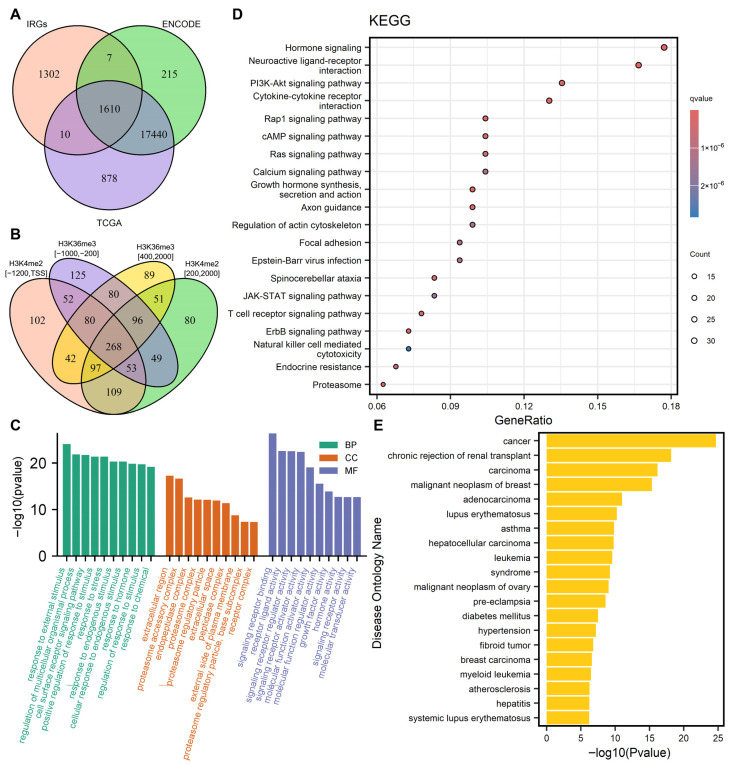
H_IRGs and their functional enrichment analysis. (**A**) Intersection of all genes and IRGs downloaded from the TCGA and ENCODE databases. (**B**) Intersection of H_IRGs in four key regions. (**C**) GO functional enrichment analysis of H_IRGs. (**D**) KEGG pathway enrichment analysis of H_IRGs. (**E**) Disease ontology enrichment analysis of H_IRGs. Abbreviations: IRGs: immune-related genes, H_IRGs: histone modification-related immune-related genes, BP: biological process, CC: cellular component, MF: molecular function.

To further clarify which genes were co-regulated by the four key regions of these three key HMs, we first integrated all genes downloaded from the TCGA and ENCODE databases and performed intersection analysis with IRGs, ultimately obtaining 1610 candidate genes (Figure 4A). Subsequently, we calculated the SDHMs of these genes in the four key regions and selected the top 50% of genes with the highest-to-lowest SDHMs as candidate H_IRGs. Since HMs in different regions have synergistic regulatory effects on gene expression, we performed an intersection analysis on the candidate H_IRGs in the four key regions and ultimately obtained 268 genes with high SDHMs as H_IRGs (Figure 4B). Secondly, to systematically analyze the regulatory role of these 268 H_IRGs in the occurrence and development of BRCA, we revealed their functional characteristics and pathway associations through GO, KEGG, and DO enrichment analysis. GO analysis results showed that these genes were significantly enriched in biological processes closely related to cancer progression, including cellular responses to chemical stimuli, regulation of immune responses, and positive regulation of cell proliferation. Cellular component analysis revealed that these genes were significantly localized in the extracellular matrix, secretory granules, and proteasome complexes, suggesting that they may function through intercellular communication and protein degradation pathways. At the molecular functional level, these genes were primarily involved in cytokine receptor binding, growth factor activity, and receptor ligand activity (Figure 4C). KEGG pathway analysis further showed that these genes were significantly enriched in core pathways that drive cancer development, such as the PI3K-Akt signaling pathway, Ras signaling pathway, JAK-STAT signaling pathway, and ErbB signaling pathway, as well as pathways that indirectly promote cancer development, such as Epstein–Barr virus infection and endocrine resistance (Figure 4D). In addition, DO enrichment results confirmed that these genes were strongly associated with breast cancer and related malignancies. They were also significantly enriched in non-neoplastic diseases including lupus erythematosus and leukemia (Figure 4E). In summary, the above results collectively indicate that these H_IRGs may be closely related to the occurrence and development of BRCA.

### 3.5. Construction of Risk Scoring Model and Survival Analysis

**Figure 5:**
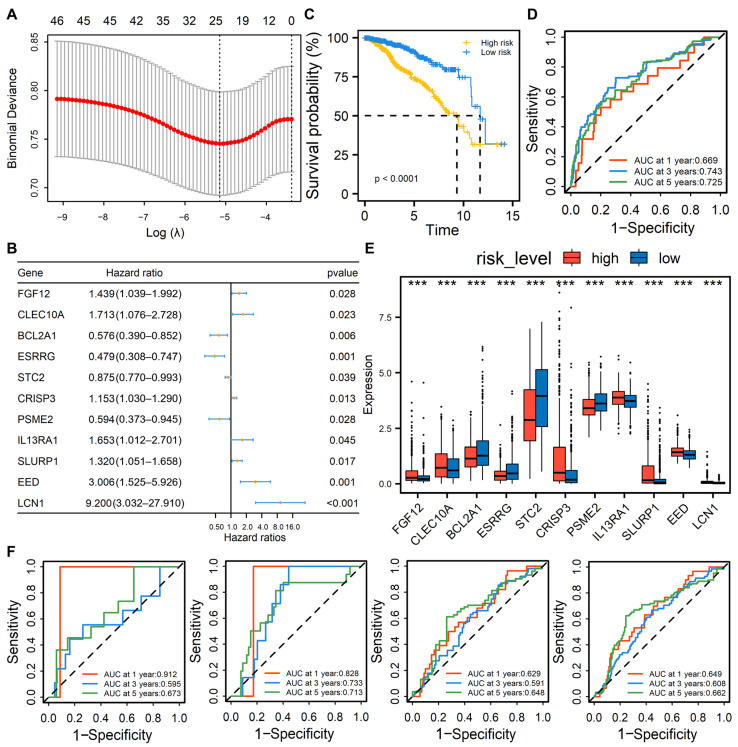
Construction of a BRCA patient risk score model and its survival analysis. (**A**) LASSO cross-validation curve (left dashed line is $\lambda_{min}=0.0058$). (**B**) Forest-plot of multivariate Cox regression analysis of 11 key IRGs. (**C**) Kaplan–Meier survival curve showing the survival probability between high- and low-risk groups. (**D**) ROC curves of the TCGA training cohort over time at 1 year, 3 years, and 5 years. (**E**) Box plot of the 11 key IRGs between high- and low-risk groups. *** *p* < 0.001. (**F**) ROC curves of the three datasets of the GEO validation cohort and their combined dataset over time at 1 year, 3 years, and 5 years. Abbreviations: BRCA: breast cancer, IRGs: immune-related genes, ROC: receiver operating characteristic curve.

To further evaluate the impact of 268 H_IRG expression on BRCA patient prognosis, we integrated the expression data of these genes with the patients’ clinical information. First, univariate Cox regression analysis was performed, in which the expression of 47 H_IRGs is significantly associated with BRCA patients’ survival (Table S3, p<0.05). Subsequently, LASSO regression analysis was used to screen these genes for characteristics. Based on the minimum λ value (Figure 5A, $\lambda_{\min}=0.0058$), 25 H_IRGs are identified. Then, multivariate Cox regression analysis was performed on these 25 genes, and the results showed that 11 H_IRGs were significantly associated with patient prognosis (p<0.05), namely, key IRGs. Our multivariate Cox regression analysis of these 11 key IRGs revealed that *BCL2A1*, *ESRRG*, *STC2*, and *PSME2* had hazard ratios (HRs) less than one and could be considered protective factors, while the remaining genes had HRs greater than one and were considered risk factors (Figure 5B). In addition, based on the multivariate Cox regression coefficients, we constructed the following risk score model:

RS = 0.364 × *FGF12* + 0.538 × *CLEC10A* − 0.551 × *BCL2A1* − 0.735 × *ESRRG* − 0.134 × *STC2* + 0.142     × *CRISP3* − 0.522 × *PSME2* + 0.503 × *IL13RA1* + 0.278 × *SLURP1* + 1.101 × *EED* + 2.219 × *LCN1*

Based on this model, we performed risk assessment on all BRCA patients and divided them into high- and low-risk groups using the median risk score (0.9105). KM survival analysis revealed that patients in the high-risk group had significantly lower survival rates and overall survival times than those in the low-risk group (Figure 5C, p<0.0001). Furthermore, we used this risk scoring model to predict patients’ survival. The results showed that the AUC values for predicting the 1 year, 3 years, and 5 years survival rates of BRCA patients reach 0.669, 0.743, and 0.725, respectively (Figure 5D). This indicates that the model has certain effectiveness and reliability in predicting patient survival and can provide a reference for clinical judgment.

In addition, we also focused on 11 key IRGs to explore their expression differences between high- and low-risk groups. The analysis results showed that the expression levels of four protective factors in the high-risk group were significantly lower than those in the low-risk group (Figure 5E, p<0.001), and the expression levels of the remaining seven risk factors in the high-risk group were significantly higher than those in the low-risk group. Therefore, we divided the patients into a high-expression group and a low-expression group according to the optimal grouping based on the expression of 11 key IRGs, and performed KM survival analysis on each group. The results showed that the survival rate of patients in the high-expression group of the four protective factors was significantly higher than that in the low-expression group (Figure S1, p<0.05), which further confirms the important impact of these IRGs on patient prognosis and provides a reference for subsequent in-depth research on the immune mechanism and treatment strategy of BRCA patients.

To further validate the predictive performance of the risk scoring model on the survival of BRCA patients, we applied it to multiple external validation cohorts from GEO. In the GSE48390 dataset, the model predicts AUC values of 0.912, 0.595, and 0.669 for 1 year, 3 years, and 5 years survival rates, respectively. The validation results for the GSE20711 dataset showed that the corresponding AUC values were 0.828, 0.733, and 0.713, respectively. In the GSE25055 dataset, the AUCs were 0.629, 0.591, and 0.648, respectively. Subsequently, we combined these three datasets for comprehensive analysis, and the results showed that the AUC values for the model’s predicted 1 year, 3 years, and 5 years survival rates for BRCA patients were 0.649, 0.608, and 0.662, respectively (Figure 5F). The results indicate that the risk scoring model demonstrates a certain survival prediction ability in different independent cohorts, but its predictive performance varies. Specifically, in the GSE25055 dataset, the AUC value declined. This may be attributed to factors such as sample size, differences in data platforms, and batch effects. Overall, this model has some potential for application in BRCA prognostic assessment.

### 3.6. Comparison of Immune Cells and Immune Activity Between High- and Low-Risk Groups

**Figure 6:**
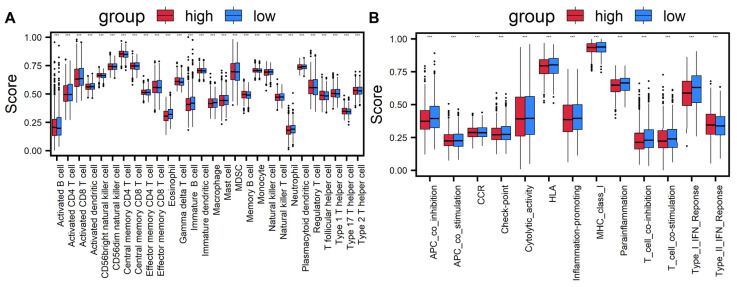
Comparison of immune cells and immune-related signaling pathways between high- and low-risk groups. (**A**) Comparison of the enrichment scores of 28 immune cells between high- and low-risk groups. (**B**) Comparison of the enrichment scores of 13 immune-related signaling pathways between high- and low-risk groups. (*** means *p* < 0.001).

To investigate the differences in immune status between high-risk and low-risk BRCA patients, we used single-sample gene set enrichment analysis (ssGSEA) to assess the status of 28 immune cells and 13 immune-related signaling pathways in different risk groups. From the results of immune cell infiltration (Figure 6A), compared to the low-risk group, the infiltration levels of immune cells such as activated B cells, activated dendritic cells, eosinophils, immature B cells, myeloid-derived suppressor cells, natural killer cells, natural killer T cells, neutrophils, and various types of T cells are significantly reduced in the high-risk group (p<0.001). This meant that the high-risk group may have relatively weak immune surveillance capabilities, thus impairing the body’s ability to recognize and eliminate tumor cells. Analysis of immune-related signaling pathways reveals that the activity of most immune-related signaling pathways is suppressed in the high-risk group, with only the type II interferon response pathway showing no decrease in activity (Figure 6B). In summary, patients in the high-risk BRCA group have poor immune status, insufficient immune surveillance capacity, and impaired activity of immune-related signaling pathways, which together create an immune microenvironment conducive to poor prognosis.

### 3.7. Drug Sensitivity Analysis of Key Immune-Related Genes

**Figure 7:**
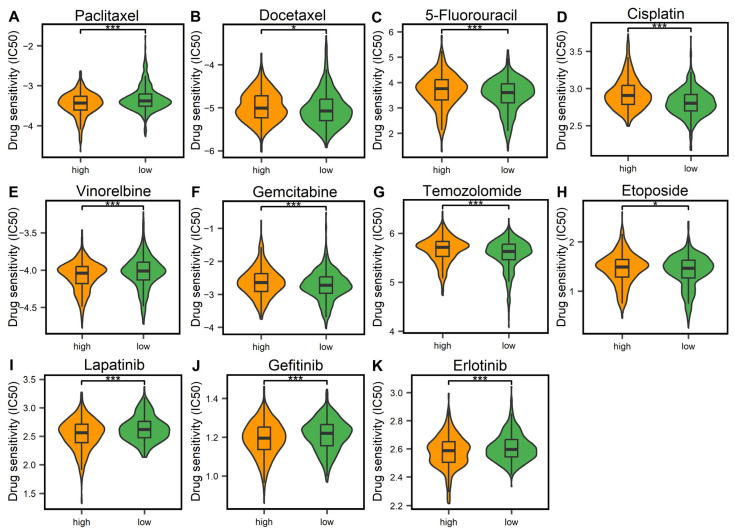
Comparison of drug sensitivity between high- and low-risk BRCA patients. (**A**–**H**) Comparison of IC50 values of commonly used BRCA chemotherapy drugs between high- and low-risk groups. (**I**–**K**) Comparison of IC50 values of BRCA targeted drugs between high- and low-risk groups (* means p<0.05, *** means p<0.001). Abbreviations: BRCA: breast cancer, IC50: half maximal inhibitory concentration.

To explore the relationship between key IRGs and the efficacy of commonly used BRCA drugs, we selected eight commonly used BRCA chemotherapy drugs (paclitaxel, docetaxel, 5-fluorouracil, cisplatin, vinorelbine, gemcitabine, temozolomide, and etoposide) and analyzed the drug responses in patients with different risk groups. The results of the drug analysis showed that patients in the high-risk group responded significantly better to paclitaxel and vinorelbine than those in the low-risk group (Figure 7A–H, p<0.001), indicating that these two drugs exhibited a stronger therapeutic effect in the high-risk group. Conversely, patients in the low-risk group were more sensitive to the remaining six drugs, including 5-Fluorouracil, while the efficacy of these drugs was relatively limited in the high-risk group. Furthermore, we identified three potential BRCA treatment drugs (lapatinib, gefitinib, and erlotinib, Figure 7I–K). The results showed that the high-risk patients were more sensitive to these three drugs. In summary, our risk model can predict chemotherapy sensitivity in BRCA patients. The 11 key IRGs in the model are expected to become potential biomarkers for assessing the sensitivity of BRCA patients to chemotherapy, and provide a reference for selecting appropriate treatment drugs for BRCA patients.

## 4. Discussion

In this study, we explored the effects of HMs on gene expression in the HMEC and MCF-7 cell lines. First, we analyzed the distribution of HM signals of differentially expressed IRGs in both cell lines and found that most HMs influenced gene expression. To investigate the specific effects of HMs on BRCA gene expression, we used the random forest algorithm to predict upregulation and downregulation of gene expression, identifying three key HMs and four key modified regions, and screening out 268 HM-related immune genes (H_IRGs). Furthermore, combining clinical data with multivariate Cox regression analysis, we screened out 11 key IRGs and constructed a risk scoring model. This model demonstrates good value in survival prognosis assessment. Finally, by exploring the relationship between these 11 key IRGs and immune infiltration and drug sensitivity, we confirmed that these genes can serve as potential biomarkers for BRCA.

As a core epigenetic regulatory mechanism, HMs play a crucial role in modulating gene expression in BRCA. Through systematic bioinformatic analysis, this study identified three key HMs and four key regions that co-regulate BRCA gene expression. Our core finding indicated that specific histone modifications, through synergistic effects, drive abnormal gene expression in BRCA. This finding echoes a growing body of research. For example, the synergistic relationship between H4K16ac and H4K20me3 has been shown to influence patient prognosis [47], while the dynamic changes in H3K79me2 and H3K36me3 are not only involved in gene expression regulation but are also considered potential therapeutic targets [48]. In addition, studies have shown that an increase in H3K36me3 leads to a decrease in H3K27me3 [49], while there is a complex interaction between H3K4me1 and H3K27ac [50]. Notably, H3K9me2 and H3K79me3 combined analysis suggest their potential value for biomarker screening and therapeutic development [51]. Although previous studies have confirmed the importance of individual modifications, such as the abnormal enrichment of the inhibitory modifications H3K9me3 and H3K20me3, which are associated with malignant phenotypes [52], as well as the roles of modifications such as H3K27ac [22,53], H3K4me2 [54], H3K4me3 [55,56], H3K79me2 [57], and H3K9ac [58,59]. The combined regulatory role of various HMs in different regions on BRCA gene expression has not yet been fully explored. Our work systematically identified specific histone modification combinatorial patterns, which not only provides a theoretical basis for understanding the co-regulation of HMs in BRCA but also offers new insights for the subsequent exploration of targeted epigenetic therapeutic strategies.

In addition, this study identified 11 key IRGs that are regulated by HMs and associated with BRCA independent prognosis. Based on their impact on BRCA prognosis, these genes are clearly divided into protective factors (*BCL2A1*, *PSME2*, *STC2*, *ESRRG*) and risk factors (*CRISP3*, *IL13RA1*, *LCN1*, *EED*, *CLEC10A*, *SLURP1*, *FGF12*). Previous studies have shown that *BCL2A1* is a key regulatory gene for treatment resistance in triple-negative breast cancer (TNBC) [60,61]. In this study, it was used as a protective factor, and its high expression was associated with better patient prognosis. High expression of *PSME2* is associated with a good prognosis of BRCA and an immune-hot tumor microenvironment (TIME) [62], which also supports its functional role as a protective factor. High expression of *STC2* is associated with a variety of cancers and is positively correlated with tumor growth, invasion, metastasis, and poor patient prognosis [63]. It is worth noting that the research results of *STC2* in the field of BRCA are inconsistent. Some studies believe that it participates in the carcinogenic process by promoting tumor growth, invasion, and metastasis, while others support its anti-cancer effect [64]. However, this study identified it as a protective factor, supporting its anticancer potential in BRCA. Currently, the research on *ESRRG* in BRCA is still unclear. Our results showed that it had a positive impact on the prognosis of BRCA, providing direction for subsequent exploration of its specific regulatory mechanism. The analysis of risk factors is also partially supported by existing studies. Overexpression of *CRISP3* in TNBC indicates poor prognosis [65,66], high expression of *L13RA1* is associated with poor prognosis in patients with invasive BRCA [67], and high expression of *LCN1* is considered an independent prognostic indicator for poor BRCA [68,69]. These results are consistent with the results of this study and verify the reliability of the screening results of this study. *EED* is highly expressed in BRCA lymph node metastasis and is associated with in situ proliferation of tumor cells [70]. It has become a potential target for tumor treatment [71,72], which further supports its driving role as a risk factor in the progression of BRCA. Notably, *CLEC10A* showed a protective effect in univariate Cox analysis, consistent with existing literature reporting that its low expression is associated with poor tumor prognosis [73,74]. However, it turns into a risk factor in a multifactorial model. This phenomenon may be partially explained by Simpson’s paradox, which is mainly caused by co-expression and confounding effects among genes. From a biological perspective, as an immune cell marker, high expression of *CLEC10A* in univariate analysis suggests immune activation; conversely, within the multivariate model, its high expression may be associated with an immunosuppressive microenvironment, thereby manifesting as an adverse prognostic factor. Furthermore, the KM survival analysis results still support its protective effect, indicating that the changes in the multivariate coefficients reflect complex interactions within the gene network, rather than *CLEC10A* itself having a pro-cancer effect. Future research is warranted to further elucidate its regulatory mechanisms within the immune microenvironment. In addition, although *SLURP1* has been widely studied in cancers such as colon cancer [75], pancreatic cancer [76], and prostate cancer [77], there are relatively few studies on BRCA. This study showed that *SLURP1* was a risk factor and its high expression is not conducive to the patient prognosis. Finally, although *FGF12* is also relatively less studied in BRCA, its study in endometrial stromal sarcoma (ESS) shows that high expression of *FGF12* is significantly associated with shortened survival time in ESS [78]. In our study, high expression of *FGF12* as a risk factor may be associated with poor prognosis in BRCA. The findings of the above studies are generally consistent with those of this study, and this study also reveals the potential impact of less-studied BRCA genes. In summary, this study revealed 11 key IRGs that are closely related to the occurrence and development of BRCA, demonstrating their value as potential molecular targets for BRCA. These findings provide new insights for BRCA research and may lay the foundation for future therapeutic strategies.

Finally, this study not only validated the potential value of commonly used chemotherapy drugs for BRCA, but also identified lapatinib, gefitinib, and erlotinib as potential therapeutic drugs with clinical potential. Among them, lapatinib has been approved for the treatment of advanced HER2-positive BRCA [79,80,81]. Although gefitinib and erlotinib are mainly used to treat lung adenocarcinoma, research on them in the BRCA field has been gradually carried out in recent years, showing potential application prospects. For example, although gefitinib faces drug resistance problems in BRCA treatment and its efficacy is limited by multiple factors [82], in the TNBC studies, the combination of gefitinib and raloxifene can prevent tumor growth and metastasis [83], and the combination with SF1126 can induce cancer cell apoptosis [84]. Similarly, erlotinib can exert a synergistic anti-cancer effect when used in combination with other drugs in TNBC studies [85,86]. These findings collectively suggest that gefitinib and erlotinib may overcome the limitations of monotherapy [87] and become potential treatment options in TNBC patients with high *EGFR* expression through specific combination regimens. This study found that lapatinib, gefitinib, and erlotinib are more sensitive in high-risk patients and may have a better therapeutic effect, but the specific mechanisms of action still need to be further explored. In summary, the three potential therapeutic agents identified in this study based on drug sensitivity analysis of 11 key IRGs not only cover clinically validated targeted drugs but also include candidate drugs with application potential, providing new insights for clinical treatment decisions for BRCA patients, especially high-risk groups.

It is worth noting that this study has several inherent limitations. First, all ChIP-seq data for histone modification analyses were retrieved exclusively from the MCF-7 cell line, which only represents the Luminal A subtype of breast cancer. Given the substantial epigenomic heterogeneity across different breast cancer molecular subtypes, the histone modification patterns of H3K4me2, H3K27me3, and H3K36me3, as well as their regulatory roles in gene expression, might differ significantly between Luminal A and more aggressive subtypes such as TNBC [88,89]. Accordingly, the H_IRGs and prognostic risk model established based on MCF-7 cell data may not fully recapitulate the epigenetic regulatory landscape of TNBC and HER2-enriched breast cancer subtypes [90]. Future studies are required to integrate ChIP-seq data from multiple breast cancer cell lines covering different molecular subtypes, such as MDA-MB-231 (TNBC) and BT-474 (HER2-enriched), to further validate and enhance the generalizability of our findings. In addition, considering that the present study is purely bioinformatics-based, further wet-lab experiments are needed to translate these computational findings into robust biological evidence. First, ChIP-qPCR assays should be performed to verify the enrichment levels of key histone modification regions. Second, knockdown and overexpression experiments of the 11 key IRGs are required to explore their functional roles in breast cancer cell proliferation, migration, invasion, and drug sensitivity. Third, expanding the panel of breast cancer cell lines with diverse subtypes will help further refine the conclusions of this study. Collectively, these efforts will help improve the robustness, applicability, and translational value of our findings, and ultimately promote the clinical implementation of immune prognostic biomarkers in breast cancer.

## 5. Conclusions

This study employed machine learning and bioinformatics methods to conduct a multi-dimensional analysis of HMs and BRCA, covering aspects such as gene expression, enrichment analysis, clinical prognosis, immune infiltration, and drug sensitivity. The study identified 11 key IRGs co-regulated by four key regions of three key HMs. Furthermore, the study successfully predicted the sensitivity of some BRCA therapeutic drugs. These findings not only provide a theoretical basis for exploring the mechanisms of BRCA development and progression, but also identify potential biomarkers, potentially providing a basis for BRCA treatment.

## Figures and Tables

**Table 1:** Optimal HM combinations and their AUC values for each HM combination number.

Combination of Optimal HMs	AUC Value
H3K79me2	0.768
H3K4me2, H3K27me3	0.852
H3K4me2, H3K27me3, H3K36me3	0.880
H3K4me1, H3K9ac, H3K27me3, H3K36me3	0.883
H3K4me1, H3K4me2, H3K4me3, H3K27me3, H3K36me3	0.890
H3K4me1, H3K4me2, H3K9ac, H3K27me3, H3K36me3, H3K79me2	0.896
H2AFZ, H3K4me1, H3K4me2, H3K9ac, H3K27me3, H3K36me3, H3K79me2	0.894
H3K4me1, H3K4me2, H3K4me3, H3K9ac, H3K9me3, H3K27me3, H3K36me3, H3K79me2	0.892
H3K4me1, H3K4me2, H3K9ac, H3K9me3, H3K27ac, H3K27me3, H3K36me3, H3K79me2, H4K20me1	0.891
H3K4me1, H3K4me2, H3K4me3, H3K9ac, H3K9me3, H3K27ac, H3K27me3, H3K36me3, H3K79me2, H4K20me1	0.890
H2AFZ, H3K4me1, H3K4me2, H3K4me3, H3K9ac, H3K9me3, H3K27ac, H3K27me3, H3K36me3, H3K79me2, H4K20me1	0.887

Abbreviations: HMs: histone modifications, AUC: area under the receiver operating characteristic curve.

**Table 2:** 17 important regions of key HMs.

Key HMs	Important Areas
H3K4me2	[−6600, −6400] bp, [−1200, TSS] bp, [TSS, 2000] bp
H3K27me3	[−3800, −3600] bp, [−3400, −3200] bp, [−2600, −2000] bp, [−1600, −1400] bp, [−1200, −600] bp, [−400, −200] bp, [TSS, 1200] bp, [1600, 2000] bp
H3K36me3	[−2800, −2400] bp, [−2200, 2000] bp, [−1400, −1200] bp, [−1000, −200] bp, [TSS, 200] bp, [400, 2000] bp

Abbreviations: HMs: histone modifications.

## Data Availability

We have used the histone modification data, the polyA+ RNA-seq data from the ENCODE database (https://www.encodeproject.org/, accessed on 22 December 2021), the gene expression data and clinical data from the TCGA database (https://cancergenome.nih.gov/, accessed on 3 July 2024), the genomic data from the UCSC database (http:/genome.ucsc.edu/, accessed on 22 December 2021), the immune-related genes from the ImmPort database (https://www.immport.org/home, accessed on 22 December 2021), and the transcriptome and clinical data from the GEO database (https://www.ncbi.nlm.nih.gov/geo/, accessed on 4 March 2025). These data are publicly available.

## Supplementary Materials

The following supporting information can be downloaded at: https://www.mdpi.com/article/10.3390/cimb48060582/s1.

## Abbreviations

BRCA	breast cancer
HMs	histone modifications
HMEC	mammary epithelial cell line
MCF-7	breast cancer cell line
IRGs	immune-related genes
H_IRGs	histone modifications-related immune-related genes
IARC	International Agency for Research on Cancer
TNBC	triple-negative breast cancer
TSSs	transcription start sites
SDHMs	HM signal differences
AUC	area under the receiver operating characteristic curve
GO	Gene Ontology
KEGG	Kyoto Encyclopedia of Genes and Genomes
DO	Disease Ontology
RS	risk scoring
ROC	receiver operating characteristic
GDSC	genomics of drug sensitivity in cancer
IC50	half maximal inhibitory concentration
ssGSEA	single-sample gene set enrichment analysis
