# Supplementary material for: Identification and Prognostic Analysis of Immune-Related Genes Co-Regulated by Key Histone Modifications in Breast Cancer
Source: Curr Issues Mol Biol. 2026 Jun 1;48(6):582. doi: 10.3390/cimb48060582 (PMC13298358; doi:10.3390/cimb48060582)
Supplement: Supplementary file 1 [file cimb-48-00582-s001.zip › Supplementary material.pdf]

Supplementary Information for

Identification and prognostic analysis of immune-related genes co-regulated by key histone modifications in breast cancer

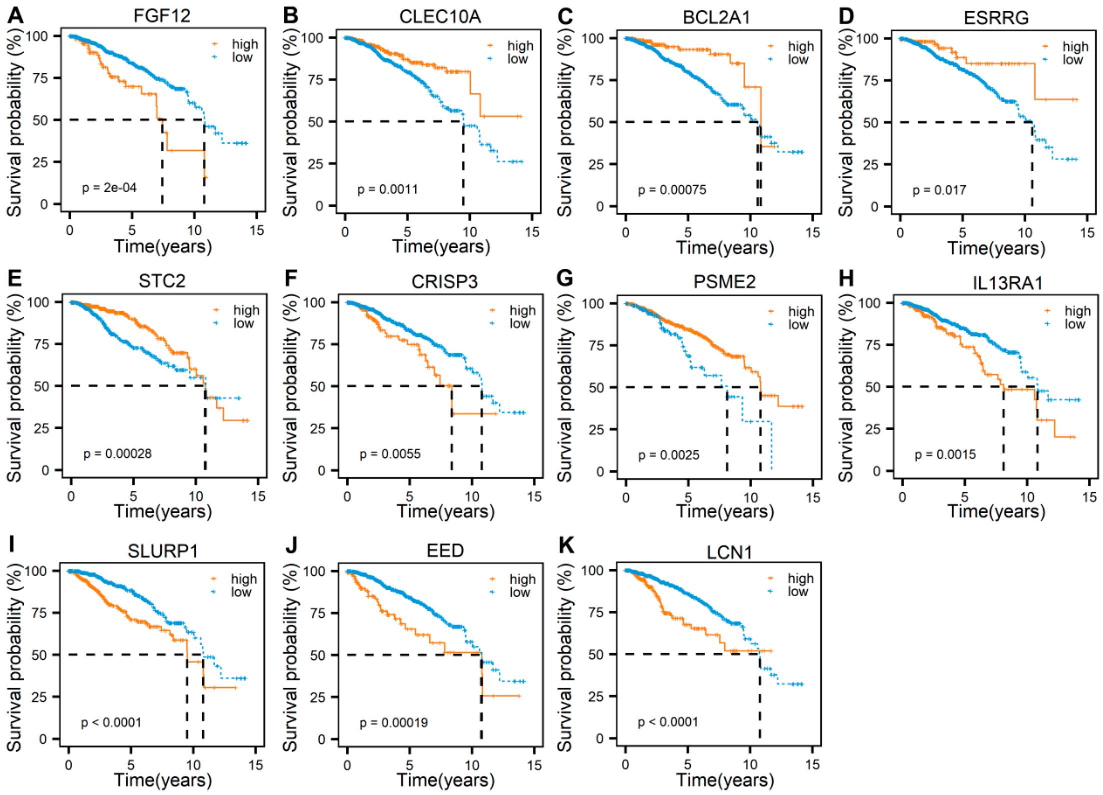

Fig. S1. Kaplan-Meier survival analysis of 11 key IRGs. A)-I) Kaplan-Meier survival curves of 11 key IRGs between high and low expression groups.
