# Supplementary material for: Identification and Prognostic Analysis of Immune-Related Genes Co-Regulated by Key Histone Modifications in Breast Cancer
Source: Curr Issues Mol Biol. 2026 Jun 1;48(6):582. doi: 10.3390/cimb48060582 (PMC13298358; doi:10.3390/cimb48060582)
Supplement: Supplementary file 1 [file cimb-48-00582-s001.zip › Table S2.pdf]

Table S2. The specific names of the twelve machine learning algorithms.

number	Algorithm Name
1	Extremely Randomized Trees (Extra Trees)
2	Random Forest
3	Decision Tree
4	eXtreme Gradient Boosting (XGBoost)
5	Naive Bayes
6	Linear Discriminant Analysis (LDA)
7	Light Gradient Boosting Machine (LightGBM)
8	Adaptive Boosting (AdaBoost)
9	Support Vector Machine with Linear Kernel (SVM-Linear Kernel)
10	k-Nearest Neighbors (k-NN)
11	Logistic Regression
12	Multilayer Perceptron (MLP)
