# Supplementary material for: Identification and Prognostic Analysis of Immune-Related Genes Co-Regulated by Key Histone Modifications in Breast Cancer
Source: Curr Issues Mol Biol. 2026 Jun 1;48(6):582. doi: 10.3390/cimb48060582 (PMC13298358; doi:10.3390/cimb48060582)
Supplement: Supplementary file 1 [file cimb-48-00582-s001.zip › Table S1.pdf]

**Table S1.** The 60 bins and its corresponding regions of the promoter region.

| bin | region（bp） | bin | region（bp） |
|---|---|---|---|
| -50 | [-10000, -9800]bp | -20 | [-4000, -3800]bp |
| -49 | [-9800, -9600]bp | -19 | [-3800, -3600]bp |
| -48 | [-9600, -9400]bp | -18 | [-3600, -3400]bp |
| -47 | [-9400, -9200]bp | -17 | [-3400, -3200]bp |
| -46 | [-9200, -9000]bp | -16 | [-3200, -3000]bp |
| -45 | [-9000, -8800]bp | -15 | [-3000, -2800]bp |
| -44 | [-8800, -8600]bp | -14 | [-2800, -2600]bp |
| -43 | [-8600, -8400]bp | -13 | [-2600, -2400]bp |
| -42 | [-8400, -8200]bp | -12 | [-2400, -2200]bp |
| -41 | [-8200, -8000]bp | -11 | [-2200, -2000]bp |
| -40 | [-8000, -7800]bp | -10 | [-2000, -1800]bp |
| -39 | [-7800, -7600]bp | -9 | [-1800, -1600]bp |
| -38 | [-7600, -7400]bp | -8 | [-1600, -1400]bp |
| -37 | [-7400, -7200]bp | -7 | [-1400, -1200]bp |
| -36 | [-7200, -7000]bp | -6 | [-1200, -1000]bp |
| -35 | [-7000, -6800]bp | -5 | [-1000, -800]bp |
| -34 | [-6800, -6600]bp | -4 | [-800, -600]bp |
| -33 | [-6600, -6400]bp | -3 | [-600, -400]bp |
| -32 | [-6400, -6200]bp | -2 | [-400, -200]bp |
| -31 | [-6200, -6000]bp | -1 | [-200, TSS]bp |
| -30 | [-6000, -5800]bp | 1 | [TSS, 200]bp |
| -29 | [-5800, -5600]bp | 2 | [200, 400]bp |
| -28 | [-5600, -5400]bp | 3 | [400, 600]bp |
| -27 | [-5400, -5200]bp | 4 | [600, 800]bp |
| -26 | [-5200, -5000]bp | 5 | [800, 1000]bp |
| -25 | [-5000, -4800]bp | 6 | [1000, 1200]bp |
| -24 | [-4800, -4600]bp | 7 | [1200, 1400]bp |
| -23 | [-4600, -4400]bp | 8 | [1400, 1600]bp |
| -22 | [-4400, -4200]bp | 9 | [1600, 1800]bp |
| -21 | [-4200, -4000]bp | 10 | [1800, 2000]bp |
