# Supplementary material for: Identification and Prognostic Analysis of Immune-Related Genes Co-Regulated by Key Histone Modifications in Breast Cancer
Source: Curr Issues Mol Biol. 2026 Jun 1;48(6):582. doi: 10.3390/cimb48060582 (PMC13298358; doi:10.3390/cimb48060582)
Supplement: Supplementary file 1 [file cimb-48-00582-s001.zip › Table S3.pdf]

Table S3. Results of univariate Cox regression analysis of 47 genes.

gene	p.value	HR	lower	upper
KIR2DL4	0.02758	0.2915 (0.09739-0.8728)	0.09739	0.8728
XCL1	0.003141	0.3967 (0.2148-0.7327)	0.2148	0.7327
TNFRSF13B	0.02889	0.3446 (0.1325-0.8961)	0.1325	0.8961
FGF12	0.008861	1.467 (1.101-1.955)	1.101	1.955
CLEC10A	0.007949	0.6778 (0.5087-0.9033)	0.5087	0.9033
ESR2	0.0156	0.002224 (1.574e-05-0.3143)	1.574e-05	0.3143
CD79A	0.001792	0.7696 (0.6529-0.9071)	0.6529	0.9071
CALM2	0.01082	1.752 (1.138-2.696)	1.138	2.696
CD27	0.006997	0.6649 (0.4943-0.8945)	0.4943	0.8945
ITGAV	0.03618	1.422 (1.023-1.976)	1.023	1.976
PAK2	0.03197	1.634 (1.043-2.559)	1.043	2.559
CD19	0.02802	0.6322 (0.42-0.9517)	0.42	0.9517
IRF9	0.04076	0.5546 (0.3153-0.9755)	0.3153	0.9755
BCL2A1	0.01085	0.7254 (0.5667-0.9286)	0.5667	0.9286
ESRRG	0.02167	0.6465 (0.4455-0.9381)	0.4455	0.9381
DPB1	0.01234	0.7427 (0.5884-0.9376)	0.5884	0.9376
MAPK9	0.0448	1.628 (1.011-2.619)	1.011	2.619
CD3G	0.006291	0.6021 (0.4184-0.8664)	0.4184	0.8664
PSMA7	0.01015	1.524 (1.105-2.1)	1.105	2.1
STC2	0.03413	0.8846 (0.7897-0.9909)	0.7897	0.9909
ADA2	0.01683	0.7244 (0.5562-0.9436)	0.5562	0.9436
CRISP3	0.001173	1.173 (1.065-1.291)	1.065	1.291
SEMA4C	0.03023	1.428 (1.035-1.972)	1.035	1.972
IL2RG	0.004248	0.6882 (0.5327-0.8891)	0.5327	0.8891
POLR2K	0.00291	1.634 (1.183-2.256)	1.183	2.256
XCL2	0.00417	0.5474 (0.3624-0.8267)	0.3624	0.8267
NCR1	0.006732	0.05093 (0.005912-0.4387)	0.005912	0.4387
TRAF1	0.006524	0.6143 (0.4324-0.8727)	0.4324	0.8727
PSME2	0.01775	0.6699 (0.481-0.9329)	0.481	0.9329
IL13RA1	0.02233	1.587 (1.068-2.358)	1.068	2.358
SLURP1	0.0008184	1.374 (1.141-1.655)	1.141	1.655
PSMD7	0.00027	2.028 (1.386-2.968)	1.386	2.968
CD37	0.03269	0.7252 (0.5401-0.9739)	0.5401	0.9739
PSME1	0.01182	0.6381 (0.4498-0.9053)	0.4498	0.9053
VPS45	0.03486	1.697 (1.038-2.774)	1.038	2.774
APOD	0.0458	0.8914 (0.7963-0.9979)	0.7963	0.9979
PIK3CB	0.01804	1.694 (1.094-2.621)	1.094	2.621
BRK1	0.02434	1.684 (1.07-2.651)	1.07	2.651
XRCC5	0.008643	2.191 (1.22-3.936)	1.22	3.936
ITGAL	0.01553	0.6721 (0.4871-0.9273)	0.4871	0.9273
PSMC6	0.04649	1.721 (1.008-2.938)	1.008	2.938
RAD21	0.008907	1.41 (1.09-1.823)	1.09	1.823
EED	0.002029	2.375 (1.371-4.113)	1.371	4.113
LCN1	0.0001383	6.83 (2.543-18.35)	2.543	18.35
CACYBP	0.001868	1.76 (1.233-2.514)	1.233	2.514
PSMD14	0.0007349	2.159 (1.381-3.375)	1.381	3.375
PIK3CA	0.002037	2.032 (1.295-3.189)	1.295	3.189
